# Supplementary material for: A Strategy for Personalized Treatment of iPS-Retinal Immune Rejections Assessed in Cynomolgus Monkey Models
Source: Int J Mol Sci. 2020 Apr 27;21(9):3077. doi: 10.3390/ijms21093077 (PMC7247695; doi:10.3390/ijms21093077)
Supplement: Supplementary file 1 [file ijms-21-03077-s001.pdf]

Supplemental Table 1.

Supplemental Table 1. Summary of the *in vivo* RPE cell transplantation in monkeys

Name	Operated eye	Graft RPE cells (human RPE)	Drugs	IHC evaluation	Rejection*	Complications
HM-1	R<br>L	Cell suspension<br>Cell suspension	IVTA<br>IVTA	6 months<br>5 months 1 week	+<br>++	None<br>None
HM-2	R<br>L	Cell suspension<br>Cell suspension	IVTA<br>IVTA	3 months 1 week<br>3 months	+<br>+++	None<br>None
HM-3	R	Cell suspension	IVTA	3 months	+	None
HM-4	R	Cell suspension	None	6 months	+	ERM
HM-5	R	Cell suspension	IVTA<br>Systemic CsA	3 months	-	None
HM-6	R<br>L	Cell suspension<br>Cell suspension	IVTA + STTA<br>IVTA + STTA	8 months 1 week<br>4 months 1 week	±<br>±	None<br>None
HM-7	R	Cell suspension	IVTA + STTA	3 months	±	Macular damage**
HM-8	R<br>L	Cell suspension<br>Cell suspension	IVTA<br>IVTA	5 months<br>4 months 2 weeks	+<br>+	ERM<br>ERM, endophthalmitis

*Grading of the RPE graft-related immune rejections was as follows: non (-), slight (±), mild (+), moderate (++), and severe (+++). **Macular damage due to operation error (reference 21 in main text). CsA – Cyclosporine A. IVTA; intravitreal triamcinolone acetonide, STTA; sub-Tenon triamcinolone acetonide, ERM; epiretinal membrane.

Supplemental Fig. 1.

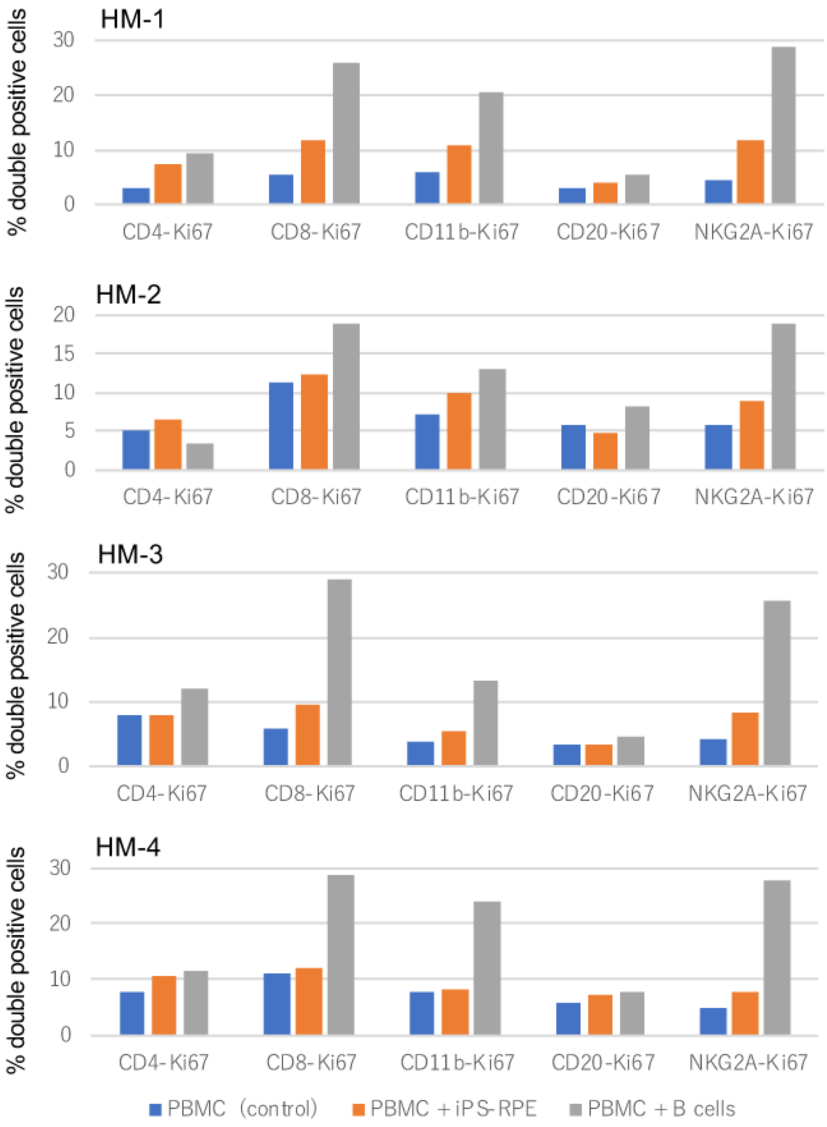

Figure S1. Summary of LGIR *in vitro* assay in monkeys.

We performed LGIR assay with PBMC from healthy monkeys, HM-1, 2, 3, 4 (n=4). PBMC were harvested and stained with anti-CD4, anti-CD8, anti-CD11b, anti-CD20, anti-NKG2A, and anti-Ki-67 antibody for FACS analysis. We then made the individual graph of Ki-67 FACS and evaluated the responses against iPS-RPE cells (e.g., CD4-Ki67 positive indicate proliferation of helper T cells). PBMC without RPE cells was experimental control (blue bars). Allogeneic B cells (B95-8 B cell lines: positive control; gray bars) were also used in this assay.

Supplemental Fig. 2.

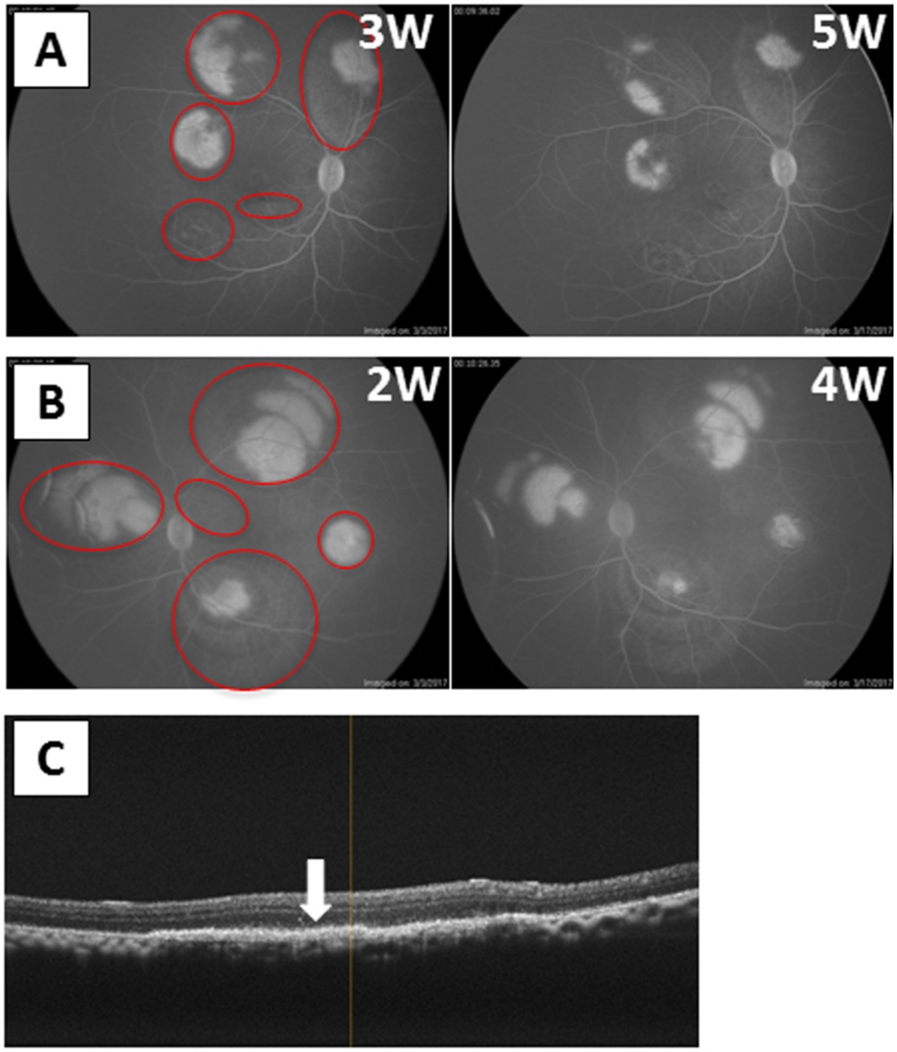

Figure S2. iPSC-RPE cell suspension transplantation displaying inflammatory differences in right and left eyes by fluorescein angiography (FA).

Late phase FA in the right and left eyes of HM-1 after transplantation are shown. Transplanted sites are shown by the red circles. (**A**) Leakages from the transplanted sites in the right eye gradually decreased. (**B**) In the nasal and upper area of the left eye, leakages remained for 4 weeks after the surgery. (**C**) OCT showed the presence of grafted cells (arrow) without any abnormal features.

Supplemental Fig. 3.

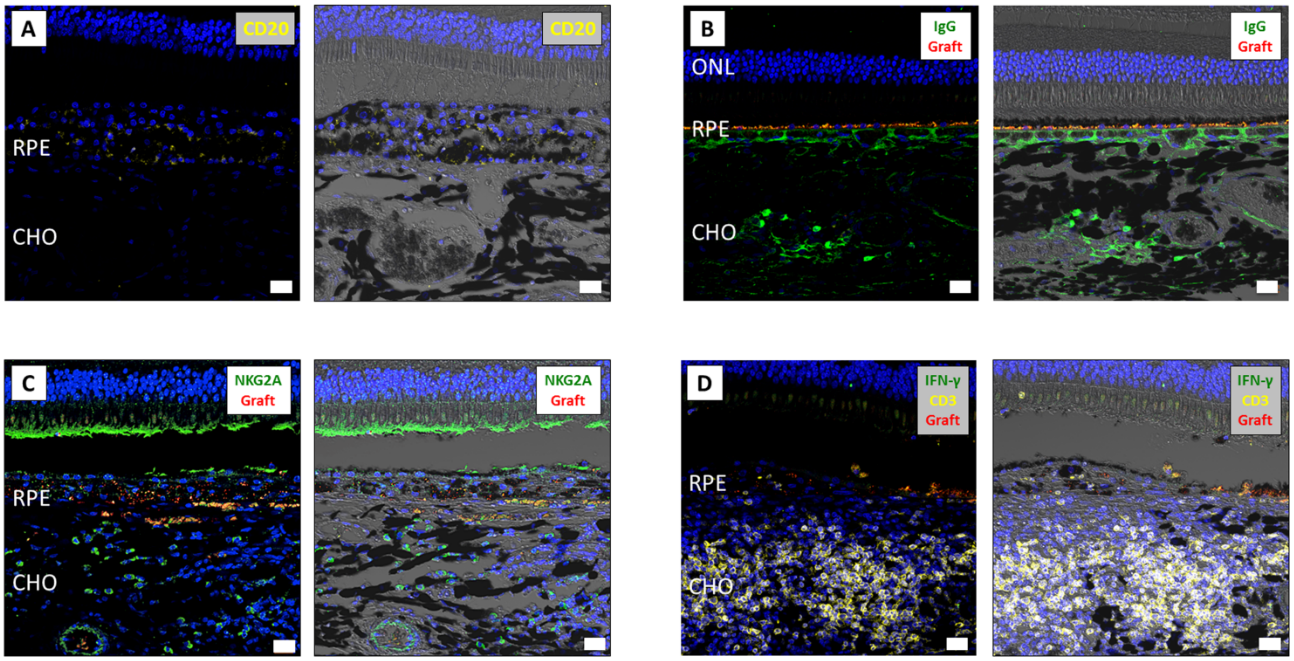

Figure S3. Representative IHC images of inflammatory immune cells in iPSC-RPE cell suspension transplantation.

(**A**) $CD20^+$ cells (B cells: Yellow) were observed around the grafts in the transplanted sites. (**B**) PKH positive graft cells extended in the subretinal space (Red: around host RPE layer). IgG deposits (Green) was also observed around the graft and in the choroid. (**C**) NKG2A positive NK cells (Green) were observed around the graft and in the choroid. (**D**) A lot of T cell infiltrates with IFN-γ (perhaps Th1 type T cell) was observed in the choroid.

Supplemental Fig. 4.

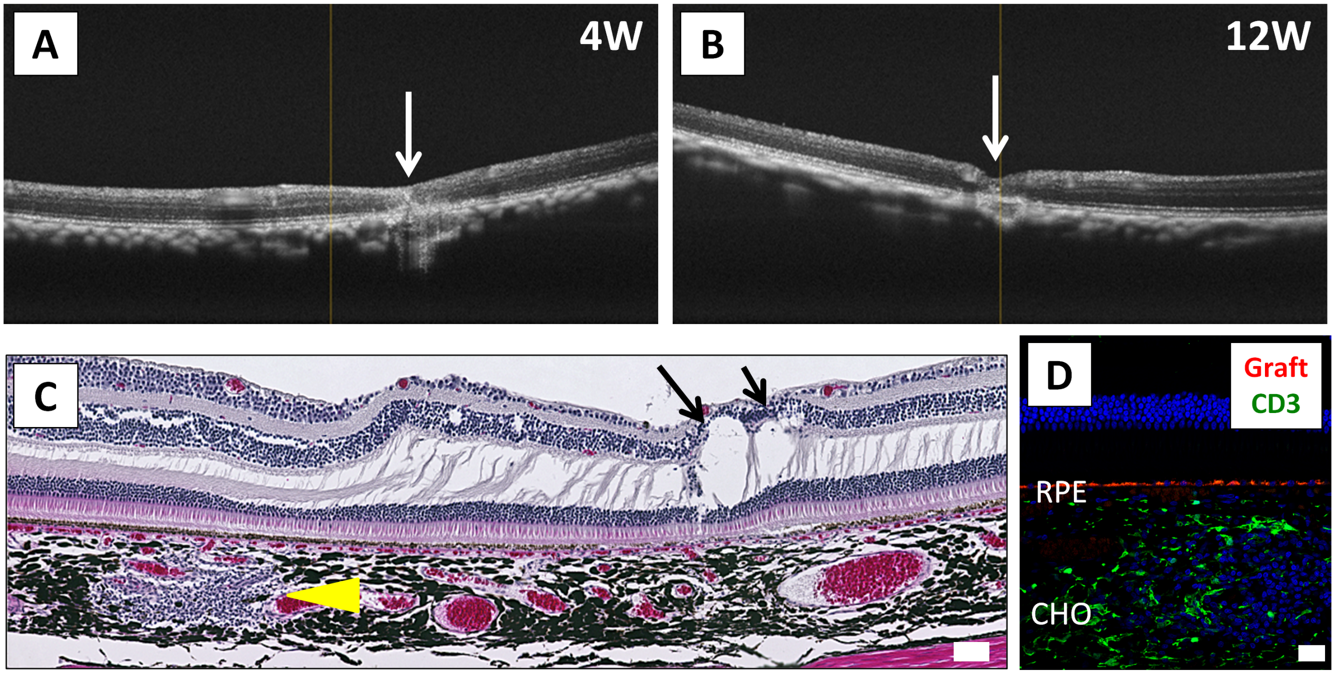

Figure S4. iPSC-RPE cell suspensions extended in the subretinal space of the transplanted monkey's eye.

(**A**) The arrow in the OCT image indicates the transplantation site. (**B**) The thickness of the host retina (arrow) decreased over time. (**C**) H&E section at 12 weeks after transplantation demonstrated cystoid retinal edema (arrows), and a mass of inflammatory cells in the choroid (arrowhead). Scale bar, 50 µm. (**D**) PKH-stained graft RPE cells were detected in the subretinal space, and CD3-positive T cells were observed in the choroid. Scale bar, 20 µm.

Supplemental Fig. 5.

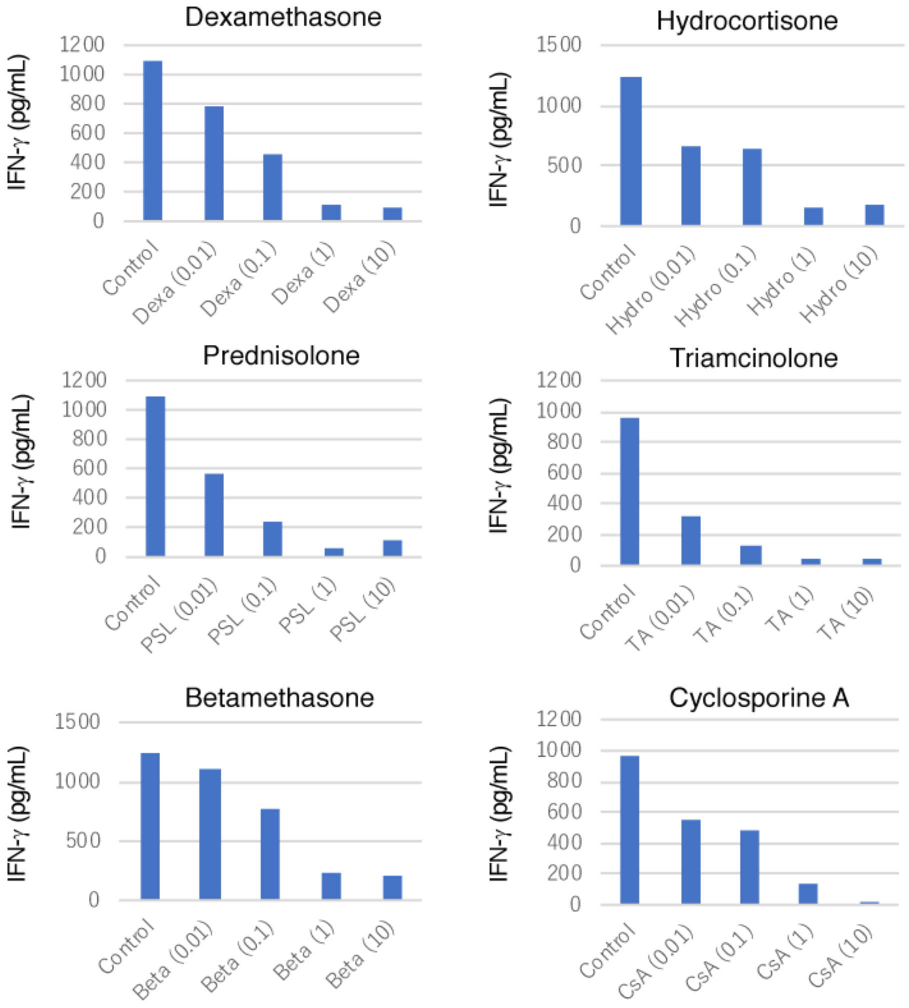

Figure S5. A range of concentrations for each drug in *in vitro* preliminary assay.

In preliminary experiments, we tested a range of concentrations for each drug before the Drug-LGIR assay. For this, we evaluated the suppression of the activities of monkey lymphocytes in PBMC (5 x 10^5/well in 96-well round plate) under 4 concentrations of each drug (0.01, 0.1, 1 and 10 μg/mL). After culturing PBMC for 5 days in the presence of the drug at each concentration, we evaluated IFN-γ levels in the supernatants. All drugs greatly suppressed the activities of lymphocytes (production of IFN-γ cytokines) at the concentration around 1 or 10 μg/mL. Therefore, we used the concentration of 1 μg/mL for all drugs in the following experiments.

Supplemental Fig. 6.

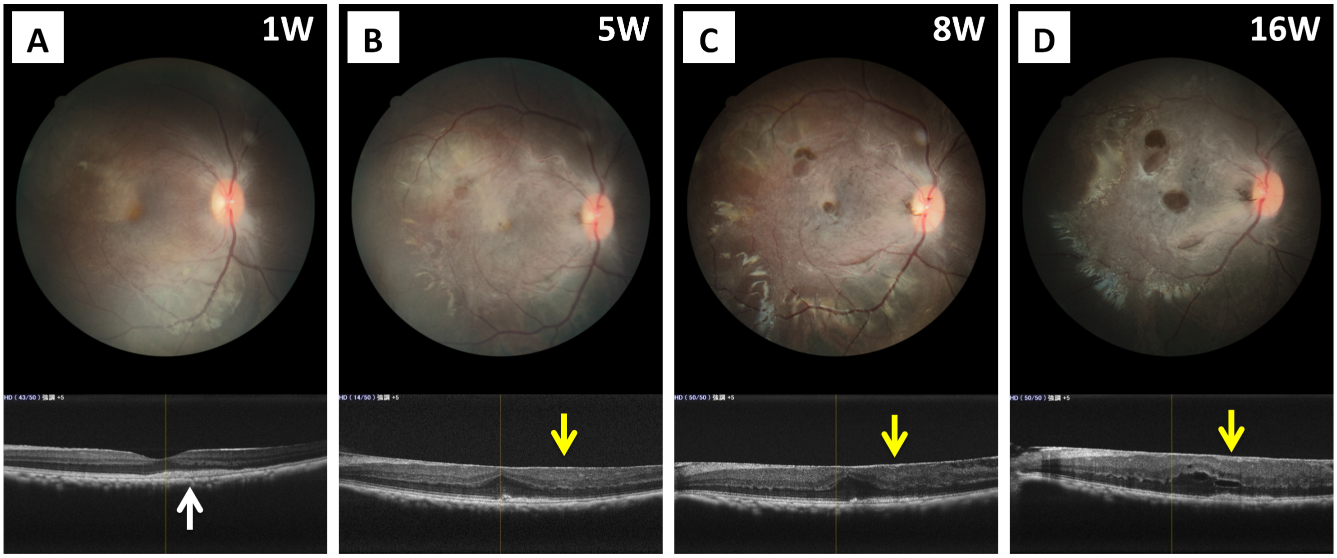

Figure S6. Surgical complications seen after the iPSC-RPE cell suspension transplantation; Epiretinal membrane.

(**A**) Grafted cells (white arrows) were detected in the subretinal space. (**B**) Epiretinal membrane (yellow arrows) was seen at 5 weeks (5W). (**C**) The thickness of the membrane increased over time up to 8W. (**D**) Retinal edema (arrowhead) can also be seen along with increases in the membrane at 16W.

Supplemental Fig. 7.

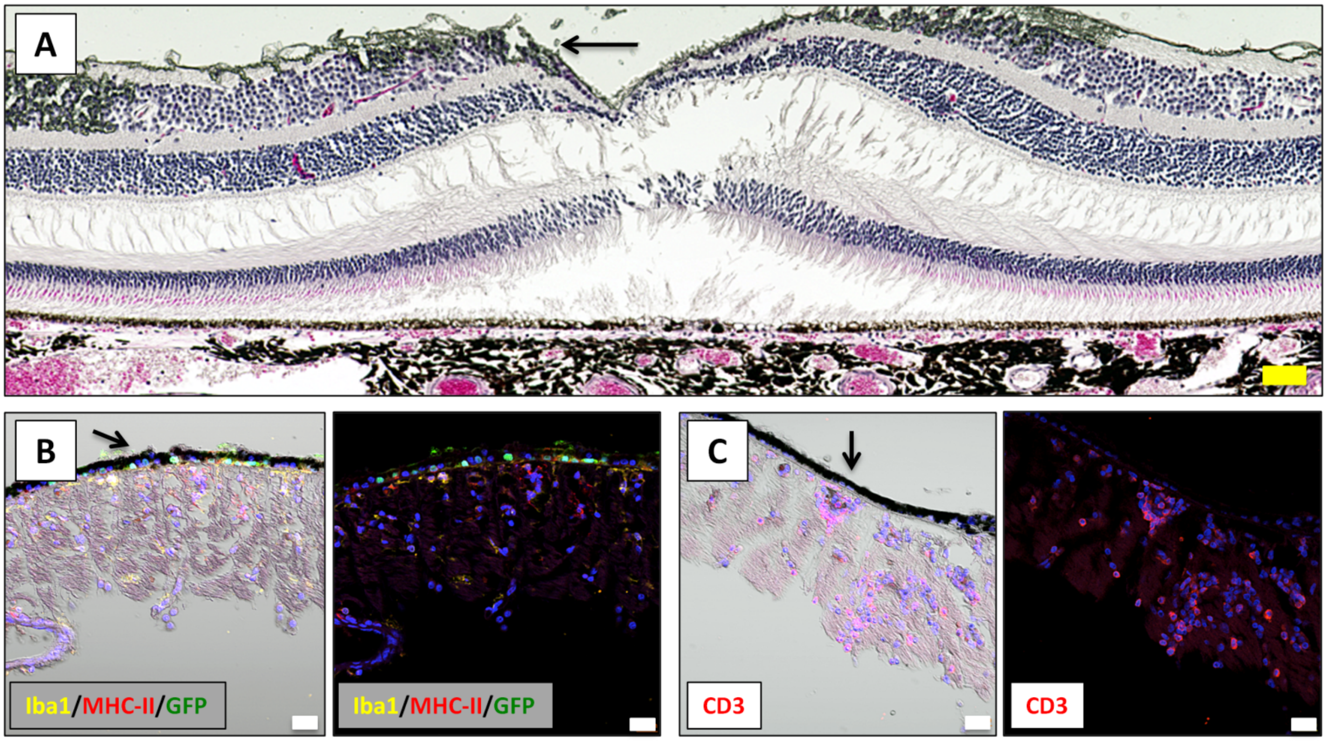

Figure S7. Surgical complication in iPSC-RPE cell suspension transplantation; epiretinal membrane (ERM) together with inflammatory cells in IHC.

(**A**) H&E stained section demonstrated the macular area and the overlying ERM (arrow). Scale bar: 50 μm. (**B**) In IHC, ERM (arrow) included GFP labeled graft RPE cells. MHC class II^{+} / $Iba1^{+}$ cells (antigen-presenting cells) were also detected in the ERM tissues. Scale bars: 20 μm. (**C**) Many $CD3^{+}$ cells (T cells) were also detected in the ERM. Scale bars: 20 μm.
